# Union rate and clinical outcomes of second-try scaphoid reconstructions after failed primary scaphoid osteosynthesis or reconstruction. A retrospective, single-center cohort study of 52 patients

**DOI:** 10.3389/fsurg.2025.1454101

**Published:** 2025-05-12

**Authors:** K. Rachunek-Medved, C. Illg, A. Einzmann, J. T. Thiel, A. Daigeler, F. Medved

**Affiliations:** Department of Hand, Plastic, Reconstructive and Burn Surgery, BG Unfallklinik Tuebingen, Eberhard Karls University Tuebingen, Tuebingen, Germany

**Keywords:** scaphoid non-union, SNAC wrist, scaphoid reconstruction, repeated scaphoid reconstruction, bone grafting, wrist

## Abstract

**Introduction:**

Scaphoid non-union after failed primary surgery presents significant therapeutic challenges.

**Methods:**

In this retrospective study, 52 patients (50 males; mean age 29.5 years) underwent secondary reconstructions (2009–2020) for proximal pole (38.5%, *n* = 20) and waist non-unions (61.5%, *n* = 32). Treatments included non-vascularized iliac crest grafts (17 patients), vascularized pedicled distal radius grafts (26), and free medial femoral condyle flaps (9). Union and scaphoid alignment were assessed by CT, while carpal alignment and arthrosis were evaluated using radiographs. Statistical analysis employed chi-square, Fisher's exact, Mann-Whitney U, and McNemar tests (R v4.4.2; *p* ≤ 0.05).

**Results:**

Union rates differed significantly between proximal pole (40%, 8/20) and waist non-unions (68.75%, 22/32; *p* = 0.04). Graft type (*p* = 0.616), osteosynthesis method (*p* = 0.827), age (*p* = 0.095), smoking (*p* = 0.582), avascular necrosis (*p* = 0.42), and prior surgeries (*p* = 0.974) showed no significant association with union. Proximal pole non-unions with AVN trended toward lower union (22.2% vs. 54.5% without AVN), though this was not statistically significant. In patients achieving union, scaphoid humpback deformity was corrected in 9/15 cases (*p* = 0.0348), and dorsal intercalated segment instability improved significantly (*p* = 0.0143). Functionally, the union group had an average extension-flexion of 112° (81% of the healthy wrist) and radial-/ulnar adduction of 40° (72% of the unaffected wrist), with grip strength averaging 42 kg (range 25.2-59.7) and a DASH score of 11 (range 0–67). The non-union group showed 114° extension-flexion (91% of the unaffected wrist) and 38° ulnar/radial abduction (78% of the healthy wrist), with grip strength averaging 46 kg (range 37.6-59.3; 89% of the unaffected wrist) and a DASH score of 10 (range 3–33).

**Discussion:**

Secondary scaphoid reconstruction demonstrates location-dependent success. The decision between secondary reconstruction, which aims to restore anatomical integrity, and salvage procedures, which prioritize predictable outcomes, hinges on balancing union potential, functional results, and patient preferences. A tailored approach remains essential to align treatment goals with individual needs.

## Introduction

According to Alshryda et al., scaphoid fractures account for about 50%–80% of carpal bones fractures (1, 2). The rate of union depends on fracture location, displacement, and time from trauma to treatment. About 10% of scaphoid fractures progress to non-union (3). The general results of primary reconstruction of scaphoid non-union remain satisfying. Pinder et al. reported in a systematic review, based on 48 publications (1.602 patients), union rates of 90% with vascularized grafts from the distal radius and an estimated union rates of 88% with the use of non-vascularized bone grafts (4).

Non-vascularized bone grafts (e.g., iliac crest or distal radius) are preferred in cases with preserved proximal pole vascularity or less complex non-unions. These grafts provide structural support and osteogenic material to promote healing but rely on adequate local blood supply, limiting their utility in avascular or long-standing non-unions (4).

Vascularized grafts (e.g., pedicled distal radius grafts) address compromised vascularity, such as proximal pole fractures or chronic non-unions, by introducing independent blood flow (5). However, they face challenges in correcting humpback deformities and managing significant bone loss, which may compromise functional outcomes compared to iliac crest grafts (6, 7).

The free medial femoral condyle (MFC) flap offers a robust solution for complex cases, combining excellent vascularization, sufficient bone stock for defect shaping, and enhanced potential for anatomical restoration (8). A notable drawback of this approach is the potential for donor site morbidity, including postoperative knee pain, although major complications are infrequent (9, 10). Additionally, the procedure is technically demanding, requiring a high level of surgical expertise and an extended operative time.

This tiered rationale—prioritizing vascular status, defect complexity, and surgeon expertise—underscores the need for individualized decision-making to optimize union rates and functional outcomes.

Even though a variety of surgical options for scaphoid reconstruction exists, the treatment of scaphoid non-union after a failed primary operation remains challenging, and the data addressing this small group of patients are very limited and mostly based on case studies. Existing studies often focus on isolated outcomes (union rates or functional scores) rather than reconciling the trade-offs between anatomical restoration, healing potential, and postoperative functionality. The union rates for second-try scaphoid reconstruction range between 50% and 91% based on studies using non-vascularized grafts and between 0% and 100% based on studies reporting on vascularized grafts (11–15). The substantial variability in reported union rates for second-attempt scaphoid reconstructions reflects the complexity of influencing factors, including patient heterogeneity, variations in surgical techniques, and the absence of prospective studies. Disparities arise from differences in bone quality, the vascular status of the nonunion site, and inconsistencies in operative methodologies, underscoring the multifaceted challenges in achieving consistent outcomes. Due to these inconsistent results of bony union reported in the literature, the surgeon faces a difficult choice between retrying a cure with a re-reconstruction or opting for well-established palliative surgeries with predictable outcomes, such as a four-corner fusion or proximal row carpectomy, which are, however, associated with a reduction in wrist motion and functional outcome.

This study aims to critically analyze the outcomes of scaphoid reconstructions following failed primary surgeries, with a particular focus on wrist function and radiological findings. By examining a comparatively large cohort of patients, it seeks to provide clinically relevant insights, addressing the existing variability in reported results and contributing to a more comprehensive understanding of the effectiveness of reconstruction techniques.

## Material and methods

**Figure 1:**
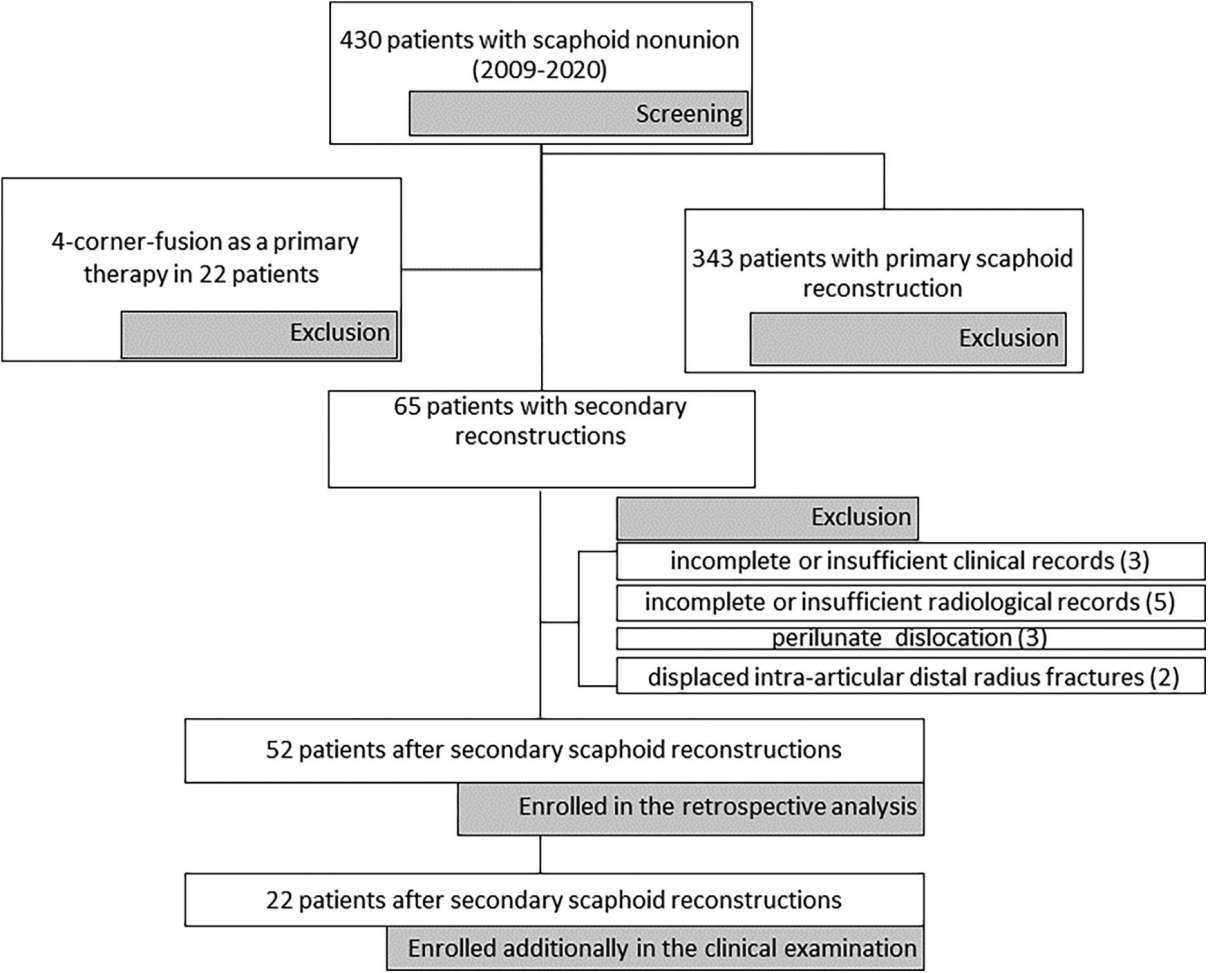
Flow-chart presenting the screening process, along with the excluded and included patients.

Approval for the retrospective study was obtained from the Institutional Review Board (Approval Number: 560/2020B0). Patients who underwent scaphoid reconstruction following failed primary surgery—including failed osteosynthesis or prior reconstruction—were included in the study. Between 2009 and 2020, we screened 430 patients treated for scaphoid non-union at our department. Of these, 343 patients were excluded due to primary scaphoid reconstructions, and an additional 22 patients were directed to salvage procedures (four corner fusion) as their primary therapy. This left 65 patients pre-selected for secondary reconstructions. Three patients were excluded due to incomplete or insufficient clinical records, and five were excluded for incomplete radiological diagnostics. Furthermore, three patients were ruled out because of complex concomitant wrist injuries (e.g., perilunate dislocation), and two were excluded due to associated radius fractures. Minimum radiological follow-up was set on at least 3 months with CT after an operation and clinical follow-up for 12 months after an operation. 52 patients met the inclusion criteria, were of legal age at the time of the study and consented to participate in the retrospective part of the study. Patients were operated on by a team of plastic surgeons, all of whom were subspecialized in hand surgery. 22 patients were willing to additionally participate in the clinical examination of the wrist and to fill in the questionnaire. 3 patients sent back the completed questionnaire without taking part in the clinical examination (Figure 1).

### Clinical records

We retrospectively analyzed the surgical records for demographic details, nicotine consumption, the type and timing of the prior operation, as well as the type and timing of the secondary scaphoid reconstruction.

Avascular necrosis of proximal fragments was diagnosed retrospectively based on surgical records, as the scaphoid bleeding is standardly described in our surgical documentation based on the findings of Green et al. (16). If there were no punctate bleeding points with tourniquet deflated, the proximal pole was considered totally avascular. MRI was not performed in this collective study due the presence of a hardware in the scaphoid of 51 out of 52 patients.

### Radiologic assessment

The radiographs and available computer tomography scans of the study-patients were retrospectively evaluated in a digital Picture Archiving and Communication System (PACS) at our institution.

The preoperative radiograms and CT scans were reviewed for the presence of scaphoid non-union, its location, alignment and eventually associated radiocarpal arthritis. The union and the time of its radiological confirmation were recorded post-operative; so were scaphoid and carpal alignment, and radiocarpal arthrosis. The assessment of radiographs and CT scans was performed by a senior hand surgeon.

### Alignment

The scapholunate angle (SLA) and radiolunate angle (RLA) were measured on lateral wrist radiographs based on the method described by Larsen et al. (17, 18). The definition of dorsal intercalated segment instability (DISI) was taken from the consensus of the International Wrist Investigators’ Workshop (19). The diagnosis of DISI was made with the wrist in neutral position identifying an RLA of at least −15° angle for the radiolunate joint. Scaphoid non-unions with a normal carpal alignment were classified as Mayo type I and those with carpal instability as Mayo type II (20).

### Osteoarthrosis

The osteoarthrosis of radiocarpal joints was evaluated on the latest radiograms of the wrist by using a modified Kellgren-Lawrence scale separately for scaphoid and lunate fossa (21) (Table 1).

**Table 1:** Kellgren-Lawrence scale of osteoarthrosis.

Grade	Description
0: Normal	
1: Questionable	Doubtful narrowing of joint space and possible osteophytic lipping
2: Mild	Definite osteophytes and possible narrowing of joint space
3: Moderate	Moderate multiple osteophytes, definite narrowing of joint space, some sclerosis, and possible deformity of bone ends
4: Severe	Large osteophytes, marked narrowing of joint space, severe sclerosis, and definite deformity of bone ends

### Humpback deformity and scaphoid malunion

The assessment of pre- and post-operative scaphoid alignment patients involved measuring the lateral intrascaphoid angle (LISA) by determining the angulation between its proximal and distal poles on sagittal CT scans. An indication of abnormal post-operative scaphoid alignment was set at a LISA measurement exceeding 45° (22–24).

### Union

The union was defined as trabecular continuity across the reconstruction site and the disappearance of the fracture gap seen on at least on four neighboring slices on 2-mm thick reconstructions in the coronal and sagittal planes defined by the long axis of the scaphoid in the CT scan with a minimal follow-up of 3 months (11). The consolidation seen or supposed on the radiograms was not considered as a validated union and, therefore, had to be confirmed with CT scans.

### Functional outcome and scores

The hand function was measured with a hand rehabilitation system by Biometrics Ltd. (EP11 system) with dedicated E-LINK computer software. The range of motion was measured with a precise electronic goniometer.

In the post-operative assessment, we used the DASH (24) and Green and O'Brien scores. Furthermore, the patients were asked to describe the rest pain of the wrist within one week prior to the examination and pain under load during examination using the Numeric Rating Scale (NRS) ranging from 0 to 10 (0: no pain, 10: intolerable pain).

### Statistical method

The distribution of metric variables among independent groups was compared using the Shapiro–Wilk test. If the assumption of normality was rejected, the Mann–Whitney *U*-test (MWU) was employed to compare two independent samples. Frequency distributions of categorical variables between independent groups were analyzed using either the chi-square test or Fisher's exact test, depending on the sample size within subgroups. The McNemar test was applied to paired categorical data to assess significant differences in the proportions of dichotomous outcomes before and after an intervention. All tests were two-sided, with statistical significance defined at a level of *p* ≤ 0.05. All analyses were conducted using the R statistical package, version 4.4.2.

## Results

Of the 52 patients, 50 were men, and only two were women. Their average age at the time of the secondary scaphoid reconstruction was 29.5 years (with a range of 15–61 years of age). In one case, the index trauma of the wrist was not remembered. 25 patients were non-smokers at the time of the secondary intervention, 17 of them were smokers, and this data was not available for 10 patients. The average time between trauma and the second operation was 23.43 months (with a range of 4.5 months to 12.91 years).

In summary, the indication was primarily made to reduce pain (in 40 patients), postpone the arthrosis of the wrist in patients with few symptoms (12 patients), and in all cases, to maintain the best possible wrist function. In 26 patients, the previous operation was performed using a cortico-cancellous bone graft from the iliac crest and a compression screw; in one patient, it was done with a pedicled vascularized bone graft from the distal radius and a K-wire, and in 25 patients, osteosynthesis with a compression screw was performed. Three patients underwent 2 scaphoid reconstructions.

In revision surgery, 17 patients underwent a bone grafting with iliac crest, supplemented with K-wire in 4 cases, with compression screw in 6 cases and without hardware (Matti-Russe) in 7 cases. Pedicled vascularized bone graft from the distal radius was used in re-reconstructions in 26 cases: in 9 cases with K-wire, in 12 cases with compression screw and in 5 cases without hardware. Reconstruction with free vascularized bone graft from the medial femoral condyle was performed in 9 patients: one without hardware, three with a compression screw and five with K-wire.

The radiological follow-ups ranged from 3 months (in 2 cases) to 153 months, with an average of 31.6 months. The clinical follow-ups for patients who participated in the clinical examination ranged from 1.2 to 13.4 years, with an average of 8.4 years.

### Union and radiological outcome

Preoperative proximal pole non-union was documented in 20 patients, accounting for 38.5% of the total 52 patients, while waist non-union was observed in 32 patients, making up 61.5% of the total. Among the 20 patients with the proximal pole lesion, complete consolidation was documented in eight patients, which represents 40% of the cases. On the other hand, among the 32 patients with scaphoid waist non-union, complete consolidation was observed in 22 patients, accounting for 68.75% of the cases. The chi-square test comparing the two groups yielded a *p*-value of 0.04.

The average time of consolidation reached 3.73 months, with a range of 2.9–8 months.

The data indicate that the median age of patients who achieved union was 23 years (Q1–Q3 = 19.5–32.5), compared to 27 years (Q1–Q3 = 22–41) for those who did not achieve union. Although patients who achieved union tended to be younger, this difference was not statistically significant (MWU, *p* = 0.095). Furthermore, the interval between the trauma and the second attempt at scaphoid reconstruction did not significantly influence the union rate (MWU, *p* = 0.2115) (Table 2).

**Table 2:** Comparison of age as well as time between trauma and secondary surgery based on union.

Patient characteristics	Union	Number	Mean	SD	Min	Q1	Median	Q3	max.	*p*-value (MWU)
Age (years)	Yes	22	26.52	9.43	15	19.5	23	32.5	53	0.095
	No	30	31.86	12.51	16	22	27	41	61	
Time between trauma and secondary surgery (months)	Yes	22	71.59	265.91	4.5	8.58	12.53	20.07	1,290	0.2115
	No	30	158.49	413.61	4.87	11.68	16.63	42.04	1,386	

status; SD, standard deviation; min, minimum; Q1, first quartile; Q3, third quartile; max, maximum; MWU, Mann–Whitney *U*-test.

The type of graft used—non-vascularized bone graft, vascularized pedicled bone graft, or free vascularized bone graft—did not have a significant impact on union outcomes (Fisher's exact test, *p* = 0.616). Similarly, the method of osteosynthesis did not show a statistically significant effect on union rates (Fisher's exact test, *p* = 0.827) (Table 3).

**Table 3:** Comparison of union outcomes after scaphoid reconstructions following failure of primary surgery based on graft type, osteosynthesis, presence of avascular necrosis, smoking status, nonunion status according to mayo classification, prior operation, and localization of nonunion, including statistical analysis and corresponding *p*-values.

Variable		*Union after secondary reconstruction*				*p*-value
		no		yes		
		*n* = 22		*n* = 30		
		number	%	number	%	
Graft type	Non-vascularized bone graft from iliac crest	6	35,29	11	64,71	0.616 (Fisher exact test)
	Vascularized pedicled bone graft from distal radius	12	46,15	14	53,85	
	Free vascularized bone graft from the medial femoral condyle	4	44.44	5	55.56	
Osteosynthesis	No	5	38,46	8	61,54	0.827 (Fisher exact test)
	Compression screw	9	42,86	12	57,14	
	K-wire	8	44,44	10	55,56	
AVN	No	15	38,46	24	61,54	0.42 (chi-square)
	Yes	7	53,85	6	46,15	
Smoking	No data	5	50	5	50	0.582 (Fisher exact test)
	No	10	40	15	60	
	Yes	7	41,18	10	58,82	
Classification of pseudoarthrosis	Mayo I	11	47,83	12	52,17	0.304 (chi-square)
	Mayo II	11	37,93	18	62,07	
Prior operation	Only osteosynthesis	11	42,31	15	57,69	0.974 (chi-square)
	Reconstruction	11	42,31	15	57,69	
Localization	Proximal pole	12	60,00	8	40,00	0.041 (chi-square)
	Scaphoid waist	10	31,25	22	68,75	

The presence of AVN did not significantly affect the likelihood of achieving union (chi-square test, *p* = 0.42), which may be attributable to the overall complexity of the cases included. Similarly, smoking status showed no significant association with union rates (Fisher's exact test, *p* = 0.582). Preoperative carpal instability, classified according to the Mayo system as Mayo I or Mayo II, also had no significant impact on union rates in this cohort (chi-square test, *p* = 0.304). Furthermore, prior surgical interventions—whether limited to osteosynthesis or involving reconstruction—did not significantly influence union rates (chi-square test, *p* = 0.974) (Table 3). Consistent results were observed in subgroup analyses of patients with proximal pole and scaphoid waist nonunions (Table 4).

**Table 4:** Comparison of union outcomes after scaphoid reconstructions following failure of primary surgery in separate groups of proximal pole and scaphoid waist nonunions based on graft type, osteosynthesis, and presence of avascular necrosis (AVN), including statistical analysis and corresponding *p*-values.

Proximal pole nonunion		Union after secondary proximal pole reconstructions				*p*-value (Fisher exact test)
		*No n* *=* *12*		*Yes n* *=* *8*		
		Number	%	Number	%	
Graft type	Non-vascularized bone graft from iliac crest	1	33.3	2	66.7	0.65
	Vascularized pedicled bone graft from distal radius	8	61.5	5	38.5	
	Free vascularized bone graft from the medial femoral condyle	3	75.0	1	25.0	
Osteosynthesis	No	3	50.0	3	50.0	1
	Compression screw	4	66.7	2	33.3	
	K-wire	5	62.5	3	37.5	
AVN	No	5	45.5	6	54.5	0.2
	Yes	7	77.8	2	22.2	
Scaphoid waist nonunion		Union after secondary scaphoid waist reconstructions				*p*-value (Fisher exact test)
		*No n* *=* *10*		*Yes n* *=* *22*		
		number	%	number	%	
Graft type	Non-vascularized bone graft from iliac crest	5	35.7	9	64.3	0.89
	Vascularized pedicled bone graft from distal radius	4	30,8	9	69,2	
	Free vascularized bone graft from the medial femoral condyle	1	20.0	4	80.0	
Osteosynthesis	No	2	28.6	5	71.4	1
	Compression screw	5	33.3	10	66.7	
	K-wire	3	30.0	7	70.0	
AVN	No	10	35.7	18	64.3	0.27
	Yes			4	100	

Descriptively, in cases of proximal pole nonunion, we observed that scaphoid reconstruction using a free vascularized bone graft from the medial femoral condyle resulted in a relatively low consolidation rate of 25%. Furthermore, in cases with AVN (avascular necrosis), 77.8% of the secondary operations failed to achieve consolidation, whereas 54.5% of cases without AVN were successful. A trend was descriptively observed regarding smoking status and the consolidation of proximal pole non-unions. In smokers, 20% of cases showed consolidation, while in non-smokers, the consolidation rate was 44.4% (Table 4). Although this difference might suggest a potential influence of smoking on the healing process, it was not statistically significant in the small patient group.

In contrast, the outcomes for patients with nonunion in the scaphoid waist appeared promising (vs. proximal pole, chi-square test, *p*-value  = 0.04), with union rates of approximately 64.3% for non-vascularized grafts, 69.2% for pedicled vascularized bone grafts from the distal radius, and up to 80% for free femoral condyle grafts (Table 4).

### Scaphoid alignment

Preoperatively, 15 patients presented with scaphoid humpback deformity (LISA > 45°). Postoperatively, the deformity was successfully corrected in 9 cases, while 6 scaphoids remained misaligned. Additionally, 2 scaphoids with preoperative LISA < 45° worsened postoperatively, and 1 developed a malunion following revision surgery. Data were missing for 3 cases. Statistical analysis (McNemar's Test, *p* = 0.0348) indicates that scaphoid humpback deformity was successfully addressed in a subset of patients.

### Carpal alignment

Preoperatively, DISI was frequently observed and classified as Stage II (unstable nonunion) according to the Mayo classification in 29 of 52 patients. Long-term correction of DISI was achieved in only 6 of these cases, while 5 patients with preoperative stable nonunion progressed to instability. All patients who demonstrated improvement in carpal alignment achieved union. The distribution of carpal misalignment pre- and postoperatively suggests that DISI was not corrected across the entire patient cohort (McNemar's Test, *p* = 0.763). However, among patients who achieved union after the most recent scaphoid reconstruction (*N* = 30), there was a significant reduction in the frequency of DISI post-revision surgery (McNemar's Test, *p* = 0.0143).

To facilitate realignment, Linscheid's maneuver was performed in 19 patients. In cases of taut pseudarthrosis without misalignment, direct stabilization was prioritized.

### Preoperative arthrosis score

The preoperative arthrosis grade in the scaphoid fossa, assessed using the Kellgren-Lawrence scale, had a mean value of 0.52 (range 0–2), with only two patients exhibiting grade 2 arthrosis. In contrast, no arthrosis was observed in the lunate fossa across any patients.

### Clinical outcome and postoperative arthrosis score

**Figure 2:**
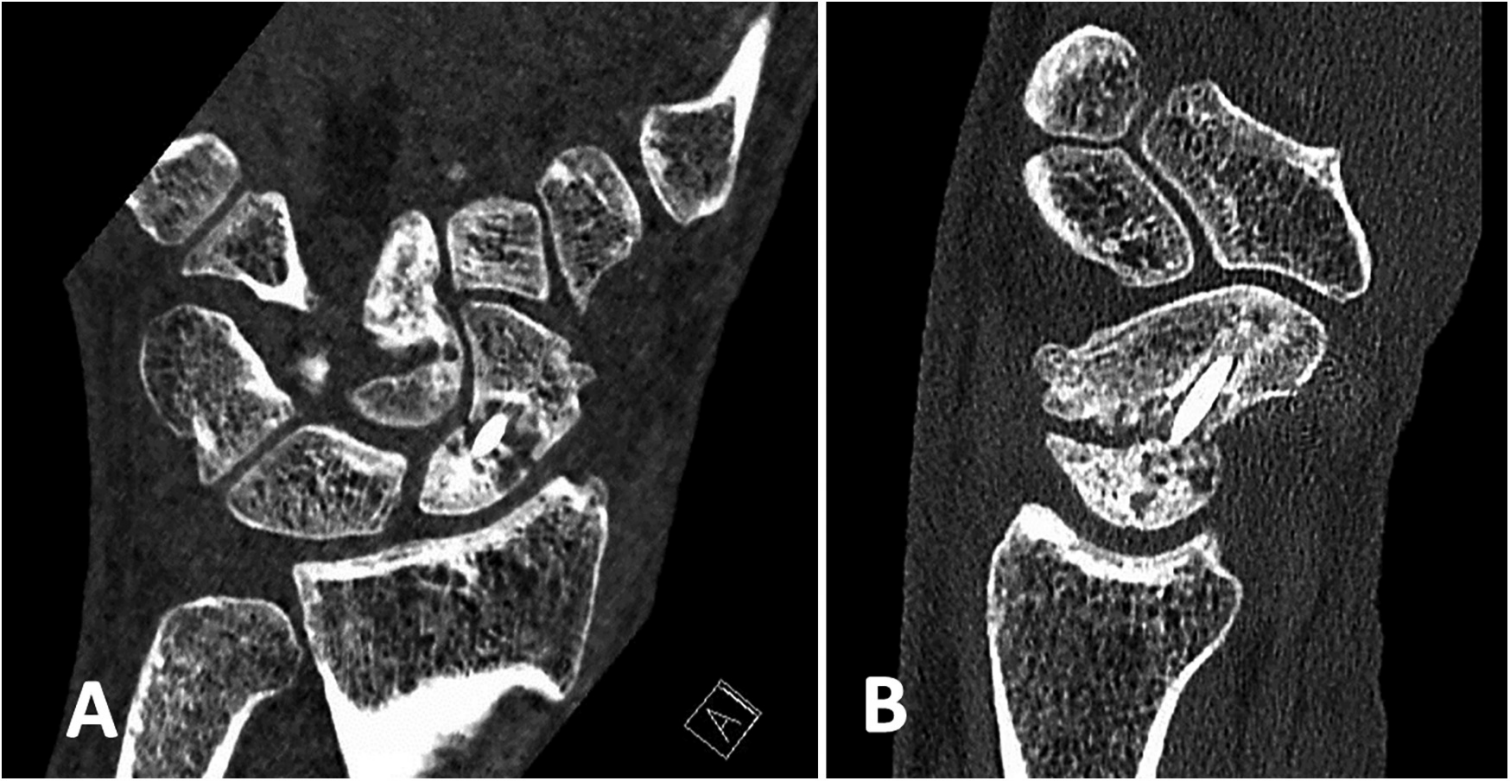
Preoperative CT scans of the patients 1.5 years after a failed osteosynthesis with compression screw showing a site of nonunion: **(A)** in coronal and **(B)** in sagittal projections. Scaphoid reconstruction has been performed (See Figure 2).

**Figure 3:**
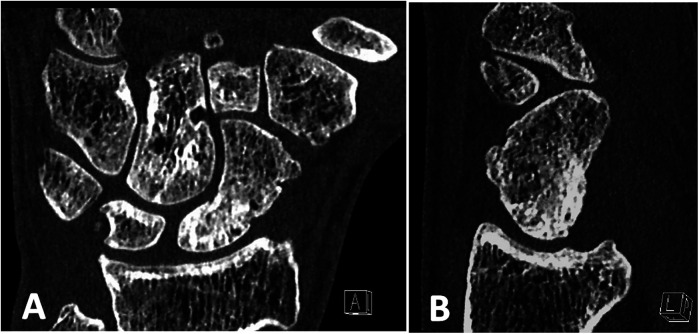
The same patient 8 months after scaphoid re-reconstruction with a cancellous bone graft from the iliac crest and vascularized bone graft pedicled on the volar carpal artery (kuhlmann), which shows a complete union of the scaphoid: **(A)** in coronal and **(B)** in sagittal projections.

**Figure 4:**
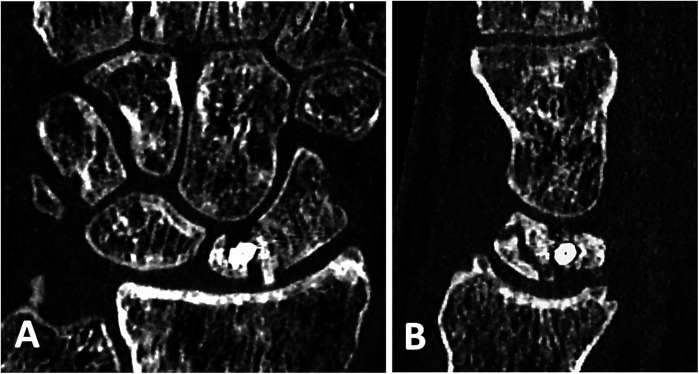
Preoperative CT scans of the patients 5 months after a failed scaphoid reconstruction with a cortico-cancellous bone graft from the iliac crest and a compression screw showing a site of nonunion: **(A)** in coronal and **(B)** in sagittal projections. Scaphoid reconstruction has been performed (See Figure 4).

**Figure 5:**
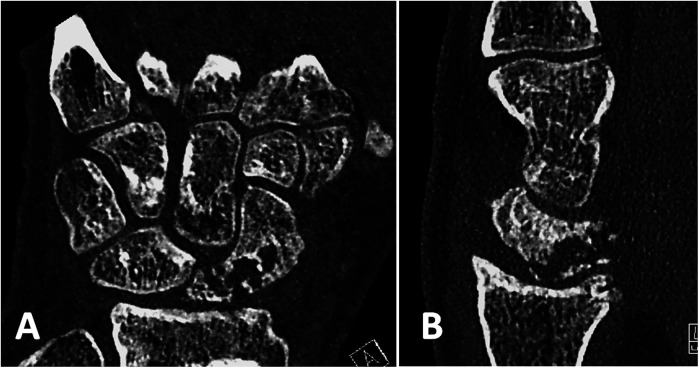
The same patient 20 months after scaphoid reconstruction with a free vascularized bone graft from the medial femoral condyle, which shows a relapse of nonunion of the scaphoid with fragmentation of the proximal pole and progression of osteoarthrosis: **(A)** in coronal and **(B)** in sagittal projections.

22 patients were willing to participate in the follow-up clinical examination of the wrist and to fill in the questionnaire. 3 patients sent back the completed questionnaire without taking part in the clinical examination. 15 patients with documented union (Figures 2, 3) and 7 patients with re-nonunion (Figures 4, 5) were examined. Due to the limited number of patients available for clinical follow-up examinations, the results have been presented descriptively.

The patients with re-nonunion were informed about the possibility of further operative treatment, such as four-corner fusion. In cases with suspected arthrosis in the lunate fossa, diagnostic arthroscopy was proposed. However, only one patient was willing to undergo an arthroscopy.

In the non-union group, the average range of motion in pronation/supination was 170° (99% of the healthy wrist), in extension/flexion 114° (91% of the healthy wrist), and in ulnar/radial abduction 38° (78% of the healthy wrist). The average complete range of motion (cROM) reached 321°, which was 93% of the unaffected wrist. The average hand grip strength reached 46 kg (range 37.6–59.3), accounting for 89% of the grip strength of the unaffected wrist.

According to the Green and O'Brien wrist score, 4 patients had good outcomes, and 3 patients had fair outcomes. The average DASH score was 10, ranging from 3 to 33. Only one patient experienced wrist pain without load and rated it at 2 on the NRS scale (ranging from 0 to 2, with an average of 0.3 for all patients with non-union). Only one patient had no pain under load. NRS under load averaged 2.4 and ranged between 1 and 5. The average postoperative arthrosis grade on the Kallgren-Lawrence scale in the scaphoid fossa was 2.25 (ranging from 1 to 3), and in the lunate fossa, it was 0.75 (ranging from 0 to 2).

In the group of patients with a successful outcome of the operation in terms of union, the average range of motion in pronation-supination reached 169°, the extension-flexion arc of motion was 112°, and the radial-ulnar abduction was 40°. These values represented 98%, 81%, and 72% of the corresponding ranges of motion of the healthy wrist, respectively. The complete range of motion averaged 321°, which represented 87% of the cROM of the unaffected wrist. Average hand grip strength reached 42 kg (ranging from 25.2 to 59.7), accounting for 87.9% of the grip strength of the unaffected wrist.

According to the Green and O'Brien wrist score, one patient had an excellent outcome, four patients recorded good outcomes, eight patients had fair outcomes, and two patients had poor outcomes. The last two patients did not have DISI deformity, and their arthrosis progressed minimally from grade 0 to grade 1 in the scaphoid fossa. However, one of them presented a complication in the form of a large exostosis following a vascularized reconstruction with a free vascularized bone graft from the medial femoral condyle. The average DASH score was 11, ranging from 0 to 67. The patient with the highest DASH score and strongest pain under load (rated 9 on NRS) suffered from a painful exostosis, as mentioned before. The pain at rest of the affected wrist was estimated to be an average of 0.3 on the NRS scale, ranging between 0 and 3. Twelve patients had no pain at rest. Pain under load was reported by 12 patients (out of 15), ranging between 0 and 9 on the NRS scale, with an average of 3.5.

The average arthrosis grade on the Kallgren-Lawrence scale in the scaphoid fossa reached 0.75 (ranging from 0 to 2), and in the lunate fossa, it was 0.06 (ranging from 0 to 1).

## Discussion

We conducted a thorough analysis of the outcomes from our study, which focused on a population of patients who underwent scaphoid revision surgery. This type of sample is rarely studied, making our findings particularly valuable. The study revealed that only 40% of surgical revisions for proximal pole non-union succeeded in terms of union, while secondary reconstructions in the scaphoid waist showed a higher success rate of 68.75%. This significant difference (*p* = 0.04) aligns with our clinical experiences. In cases of proximal pole non-unions with AVN, the union rate was descriptively even lower as it was achieved in only 22.2% of patients. Interestingly, vascularized grafts showed descriptively more favorable results in the reconstructions of scaphoid waist non-unions, which was not observed in cases of proximal pole non-unions. This difference may be attributed to the coexistence of severe AVN in the latter group.

For cases of successful scaphoid reconstructions after failure of primary surgery with documented union, the clinical outcomes were favorable, with cROM reaching 87% of the healthy wrist and hand grip strength accounting for 87.9% of the unaffected hand. Surprisingly, the clinical outcomes of patients with nonunion after secondary surgery were descriptively similar (cROM reaching 93% and hand grip strength accounting for 89% of the unaffected wrist, respectively). However, the arthrosis grade in the scaphoid and lunate fossa was descriptively higher in this group, potentially leading to worsening conditions in the long term. The favorable clinical outcomes in terms of ROM and grip strength, as well as lower arthrosis rates support the decision for scaphoid re-reconstruction in this very young and active study population, with an average age of 29.5 years old.

According to the meta-analysis conducted by Merrell et al., there is a trend towards improved union rates with vascularized grafts over non-vascularized grafts with screw fixation (94% union in 35 patients vs. 81% union in 26 patients) in case of revision surgeries of the scaphoid (25). These findings appear to align with our observations in the case of re-reconstructions of waist non-unions. However, in the cited meta-analysis, there was no differentiation between the region of the lesion, which, in our opinion, can influence the union rate even more than the operating technique does. That meta-analysis included studies that used vascularized grafts for treatment of previously failed surgeries in the case of 5 or fewer patients with a range of union from 0% to 100% (12–14, 26–29). Another study conducted by Preisser et al. examined 25 patients with a relapse of non-union after previous reconstruction with cancellous bone graft (with or without fixation screw) (15). The patients were treated with repeated non-vascularized iliac crest grafts (10 with hardware and 15 without according to Matti-Russe technique) (15). The authors reported a 64% union rate, but did not report on the exact localization of non-union. Furthermore, a long-term study was conducted by Reigstad et al., who examined 18 patients with a recurrence of non-union after previous non-vascularized reconstructions (30). Eleven of the non-unions were in the middle, and seven were in the proximal third of the scaphoid. The authors reported that 16 of the 18 non-unions healed (88.8%); however, in two cases, a third attempt was necessary to achieve consolidation. The mean DASH was 18 and VAS score reached 21/100 respectively. The data regarding the patients’ reported outcomes are slightly worse than ours (DASH 11), but the difference could result from a longer follow-up by Reigstad et al. (min. 8 y.). Bynum et al. conducted a study on a cohort of 15 patients who had undergone repeated Russe bone grafting following previous procedure failures (31). The results revealed that eight out of the 15 patients (53%) achieved union. Notably, the study comprised a high percentage (86.7%) of waist non-unions, with only two cases involving proximal pole non-union.

The union rate in proximal pole lesions was significantly lower compared to waist lesions (*p* = 0.04). In fact, only 40% of proximal pole non-unions showed consolidation in CT scans. This raises the question of exploring alternative treatments for these patients, including salvage procedures such as four-corner fusion. Hence, a comprehensive preoperative diagnostic assessment with arthroscopy is crucial for reevaluating the cartilage damage in the wrist. Despite a larger reduction in wrist motion, the union rate after four-corner fusion appears to be more predictable. According to the meta-analysis conducted by Andronic et al., the average union rate can reach 91% (range: 76%–100%) (32). The same authors reported a conversion to total wrist fusion (TWF) in 6% of cases on average (range: 0%–20%). On the other hand, Reigstad et al. observed a higher conversion rate to TWF, reaching 33% of cases during a follow-up of 11 (4–19) years after surgery (33). Possibly the decision to undertake salvage operation instead of reconstruction in case of recalcitrant proximal pole non-union should be postponed and the indication of four-corner fusion in scaphoid non-union cases should be limited to older patients and those with lower demand.

Our study demonstrated that humpback deformity can be effectively corrected through revision surgery (*p* = 0.035), with improvements in DISI also achievable when union is attained (*p* = 0.014). This finding is particularly significant, as scaphoid malunion is associated with reduced functional outcomes, and correcting the deformity may enhance the chances of achieving union (6, 23, 34). However, addressing chronic misalignment during revision surgery remains a complex challenge, underscoring the importance of precise graft shaping. In a study on the “butterfly bone graft,” a modification of the Matti-Russe technique, researchers reported excellent outcomes, including proper repositioning and a 100% healing rate in cases of humpback deformity (35). The graft in this technique features a central axis connecting the proximal and distal poles of the scaphoid, with two wedge-shaped “wings” in the middle third to aid in correction. The lower union rates observed in our study may be explained by the inclusion of patients with prior surgical interventions, a group excluded in the “butterfly bone graft” study. Given the unique shape of the scaphoid and the critical need for precise anatomical reconstruction during surgery, further innovative approaches are being developed. These encompass a spectrum of approaches, including guiding templates for K-wire fixation planned using 3D models derived from CT scans, as well as the individualized 3D modeling of bone grafts (36–38). Schweizer et al. reported significantly improved anatomical reconstruction outcomes using a two-part reconstruction template with designated spaces for K-wire placement. This template, based on a CT-mirrored model of the contralateral scaphoid, yielded superior repositioning results compared to the traditional freehand technique (39).

Oki et al. designed a custom plate for reconstructing scaphoid nonunion in cases with pre-existing screws (40). Their approach involved preoperative planning based on a mirrored model of the unaffected scaphoid, employing surface reconstruction at the nonunion site and fabricating the plate with a three-dimensional (3D) printer. This customized plate facilitated surgery by enabling precise prediction of fragment reduction, gap size, implant positioning, and screw orientation in complex scaphoid fractures with pre-existing screws (40). Furthermore, in a biomechanical study, Mandaleson et al. evaluated various fixation techniques using a scaphoid nonunion model with bone loss. Their findings demonstrated that both plate fixation and double screw fixation provided significantly greater stability, stiffness, and energy absorption compared to a single compression screw (41).

Houdek et al. and Taylor et al. described the use of preoperative printed templates based on 3D scaphoid models for the accurate intraoperative harvesting of osteochondral free femoral condyles (37, 38). These templates ensured proper alignment of the chondral surface within the radiocarpal joint, minimizing the need for intraoperative re-contouring. Such methods have demonstrated potential to enhance repositioning outcomes, particularly in complex cases. However, these techniques have not yet been implemented in our study.

An alternative approach to improving outcomes in scaphoid waist nonunion is osteosynthesis using shape memory staples (SMS) augmented with gelled platelet-rich plasma (GPRP). De Vitis et al. reported union rates of 95.2% in the SMS-only group and 100% in the SMS + GPRP group, though the difference was not statistically significant (42). Notably, the SMS + GPRP group demonstrated significant improvements in functional outcomes, including the Mayo Wrist Score, QuickDASH score, and pain levels as measured by the visual analog scale (VAS), at three months postoperatively (*p* = 0.02). Patients with prior scaphoid surgery, gaps greater than 2 mm, scaphoid shortening, or humpback deformity were excluded from the study. In a separate study, De Vitis et al. investigated the role of GPRP in the fixation of subacute proximal pole scaphoid fractures (43). They observed union rates of 85.7% in the screw fixation-only group and 100% in the screw fixation + GPRP group, though this difference was not statistically significant. However, patients in the GPRP group achieved significantly faster healing times (2.3 months vs. 3.1 months, *p* = 0.0001) and an earlier return to work (10.4 weeks vs. 15.1 weeks, *p* = 0.0001).

Zhong et al. evaluated the effects of platelet-rich plasma (PRP) in the management of scaphoid nonunion across three groups: bone graft with screw fixation, screw fixation alone, and screw fixation augmented by PRP. Their study, which included patients with minimal sclerosis and bone resorption less than 5 mm, found that the PRP group exhibited lower VAS values and higher Mayo Wrist Scores at three months postoperatively (44). The patient cohort in our study presented with more challenging nonunions, including prior surgical interventions, rendering these described methods less applicable. Nevertheless, the additional application of PRP to classical reconstruction with bone grafts could potentially serve as a valuable adjunct. To date, only small case series have been conducted in this context, highlighting the need for further investigation (45).

When considering revision surgery, it is essential to assess also classical risk factors for scaphoid non-union after reconstruction, such as AVN. Ditsios et al. evaluated healing rates for vascularized grafts (1,2- Intercompartmental Supraretinacular Artery-Based Vascularized Graft) in case of proximal pole AVN and showed that the union was 2,91 times more probable when there wasn't proximal pole AVN and 10,06 times more probable for non-smokers (46). In the newest metanalysis of Duncumb et al. the consolidation rate was also significantly greater in studies, which excluded the proximal pole non-unions and these with AVN (95,6% vs. 86,8%) (47). No significant difference was observed in union rates between vascularized and non-vascularized grafts and between any of the fixation techniques used in the included studies. The method of osteosynthesis from the primary procedure additionally impact the potential choices for the second intervention (such as osteolysis around a failed internal screw), which may have resulted in the lower rates of bony consolidation. The lower union rates observed in our study can be attributed to the fact that we specifically focused on revision surgeries and included a high percentage of cases with AVN. Furthermore, in certain cases, no hardware (25% of cases), or only K-wire (34.6%) was used in the secondary reconstructions.

Although our analysis of a small patient cohort did not demonstrate a statistically significant effect of smoking on scaphoid nonunion, descriptive observations revealed a consolidation rate of 20% in smokers compared to 44.4% in non-smokers for proximal pole nonunions. Reports based on larger patient populations indicate that smoking negatively impacts union rates in scaphoid nonunions (34, 46, 48, 49). Konstantinidis et al., in their meta-analysis of 18 studies comparing 335 smokers to 136 non-smokers, found that scaphoid non-union healing was significantly more likely in non-smokers (OR = 5.54, *p* < 0.001) (50). However, they did not observe an effect of smoking cessation on union rates. In contrast, Iwamae et al. investigated the influence of extracts from combustible cigarettes in an *in vivo* rat model (51). They reported significantly higher cortical bone mineral density (*p* = 0.013), content (*p* = 0.013), and a higher bone union score (*p* = 0.046) at the fracture site in the cessation group.

The study has limitations inherent to its retrospective design and small cohort, given the rarity of revision scaphoid surgery. First, surgical heterogeneity—including variable fixation (K-wires, screws, no hardware) and graft choices (vascularized vs. non-vascularized)—likely influenced outcomes, as standardized protocols for revision techniques are lacking. Second, selection bias may exist, as patients undergoing reconstruction (vs. salvage procedures) may have had better baseline vascularity or simpler defects, limiting generalizability. Third, the impact of surgeon learning curve was unaddressed; outcomes may improve with experience in complex techniques like vascularized grafts. Moreover, the pre-operative functional data was limited, and there were instances of missing NRS scores regarding pain, making it impossible to directly compare the condition before and after surgery. Additionally, not all patients received clinical follow-up after the surgery, and the duration of clinical follow-up might have been relatively short in some cases (minimum 1.2 years; average 8.4 years) to effectively demonstrate the differences between the groups with achieved union and those with failed reconstruction.

The outcomes of scaphoid reconstruction following a failed prior surgical attempt are primarily influenced by the location of the non-union, with significantly lower union rates observed in cases involving the proximal pole. Due to the lower union rates associated with proximal pole non-unions, the decision to pursue secondary scaphoid reconstruction should be carefully individualized for each patient. For individuals presenting with additional risk factors for scaphoid non-union—such as smoking, avascular necrosis (AVN), or low functional demands—alternative wrist salvage procedures like four-corner fusion, which offer more predictable consolidation rates, should be considered and discussed. Prospective studies comparing long-term outcomes (e.g., arthritis progression, patient-reported function) of secondary reconstruction vs. early salvage procedures (e.g., four-corner fusion) in proximal pole non-unions could refine decision-making paradigms. Future studies should adopt uniform metrics for reporting outcomes (e.g., union criteria, functional scores) to facilitate meta-analyses and reduce heterogeneity in evidence synthesis. The role of adjunctive therapies, such as Platelet-Rich Plasma warrants further exploration in this context. While preliminary studies have demonstrated that PRP may enhance bone healing and improve functional outcomes in select groups of patients, its efficacy in more challenging cases, such as those with AVN or extensive bone resorption, remains unclear. Future clinical trials should evaluate the benefits of PRP application when combined with traditional reconstruction techniques, such as vascularized bone grafts or advanced fixation methods. Future research should focus on further validating the efficacy of 3D modeling and custom templates in improving outcomes for scaphoid reconstruction, particularly in complex nonunion cases. Comparative studies evaluating these innovative techniques against traditional methods could provide valuable insights into their advantages in terms of anatomical accuracy, union rates, and functional recovery. Additionally, integrating these approaches with adjunctive therapies, such as PRP or bioengineered grafts, may offer synergistic benefits and should be explored. Finally, efforts to streamline the accessibility and cost-effectiveness of these advanced technologies will be critical for their broader clinical adoption.

## Data Availability

The raw data supporting the conclusions of this article will be made available by the authors, without undue reservation.
